# Whole exome sequencing of well-differentiated liposarcoma and dedifferentiated liposarcoma in older woman: a case report

**DOI:** 10.3389/fmed.2023.1237246

**Published:** 2023-08-15

**Authors:** Zidan Zhao, Xiaoyan Chen, Jie Xu, Yuntao Shi, Tsz Kin Mak, Mingyu Huo, Changhua Zhang

**Affiliations:** ^1^Digestive Diseases Center, The Seventh Affiliated Hospital of Sun Yat-sen University, Shenzhen, China; ^2^Department of Pathology, The Seventh Affiliated Hospital of Sun Yat-Sen University, Shenzhen, Guangdong, China; ^3^Guangdong Provincial Key Laboratory of Digestive Cancer Research, The Seventh Affiliated Hospital of Sun Yat-sen University, Shenzhen, Guangdong, China

**Keywords:** retroperitoneal tumor, well-differentiated liposarcoma, dedifferentiated liposarcoma, ROS1-related fusion, whole-exome sequencing

## Abstract

**Background:**

Common kinds of soft tissue sarcomas (STS) include well-differentiated liposarcoma (WDLPS) and dedifferentiated liposarcoma (DDLPS). In this case, we present a comprehensive clinical profile of a patient who underwent multiple recurrences during the progression from WDLPS to DDLPS.

**Case presentation:**

A 62-year-old Asian female underwent retroperitoneal resection of a large tumor 11 years ago, the initial pathology revealed a fibrolipoma-like lesion. Over the next six years, the patient underwent three resections for recurrence of abdominal tumors. Postoperative histology shows mature adipose tissue with scattered “adipoblast”-like cells with moderate-to-severe heterogeneous spindle cells, pleomorphic cells, or tumor giant cells. Immunohistochemistry (IHC) demonstrated positive staining for MDM2 and CDK4, confirming that the abdominal tumor was WDLPS and gradually progressing to DDLPS. Post-operative targeted sequencing and IHC confirmed the POC1B::ROS1 fusion gene in DDLPS. Whole-exome sequencing (WES) revealed that WDLPS and DDLPS shared similar somatic mutations and copy number variations (CNVs), whereas DDLPS had more mutated genes and a higher and more concentrated amplification of the chromosome 12q region. Furthermore, somatic mutations in DDLPS were significantly reduced after treatment with CDK4 inhibitors, while CNVs remained elevated.

**Conclusion:**

Due to the high likelihood of recurrence of liposarcoma, various effective treatments should be taken into consideration even if surgery is the primary treatment for recurrent liposarcoma. To effectively control the course of the disease following surgery, combination targeted therapy may be a viable alternative to chemotherapy and radiotherapy in the treatment of liposarcoma.

## Background

Liposarcoma (LPS) is the most prevalent soft tissue sarcoma, accounting for approximately 20% of all mesenchymal tissue malignancies (1), and can be subdivided into five subtypes (2): atypical lipomatous tumor/well-differentiated liposarcoma (ALT/WDLPS), DDLPS, myxoid liposarcoma (MLPS), pleomorphic liposarcoma (PLPS), and myxoid pleomorphic liposarcoma (MPLPS), each with unique morphology, histology, natural course, and clinical behavior. Patients with pure WDLPS have a five-year disease-specific survival rate of 93%, compared to 44% for those with DDLPS (3). Clinically, DDLPS represents the progression of WDLPS from an indolent, sometimes locally aggressive lesion to a more rapidly growing disease with metastatic potential, which is now thought to be due to genomic alterations DDLPS is more complex than WDLPS (4). The location of tumorigenesis is the most significant negative prognostic factor for WDLPS and DDLPS. Retroperitoneal WDLPS and DDLPS have a higher rate of recurrence and a worse survival rate compared to other locations (5). Retroperitoneal DDLPS is characterized by a substantially greater local recurrence rate, and patients are frequently had to undergo several surgeries that become increasingly challenging. Understanding the hereditary molecular features of DDLPS and WDLPS is beneficial for adjuvant medication therapy. Genetically, WDLPS and DDLPS share the same fundamental genetic aberration, which consists of amplified sequences starting from the long arm of chromosome 12 (6). We provided a comprehensive history of recurrent retroperitoneal liposarcoma in an elderly woman, as well as a summary of the changes in clinical and molecular genetic characteristics that accompany disease development and targeted treatment.

## Materials and methods

### Case presentation

**Figure 1:**
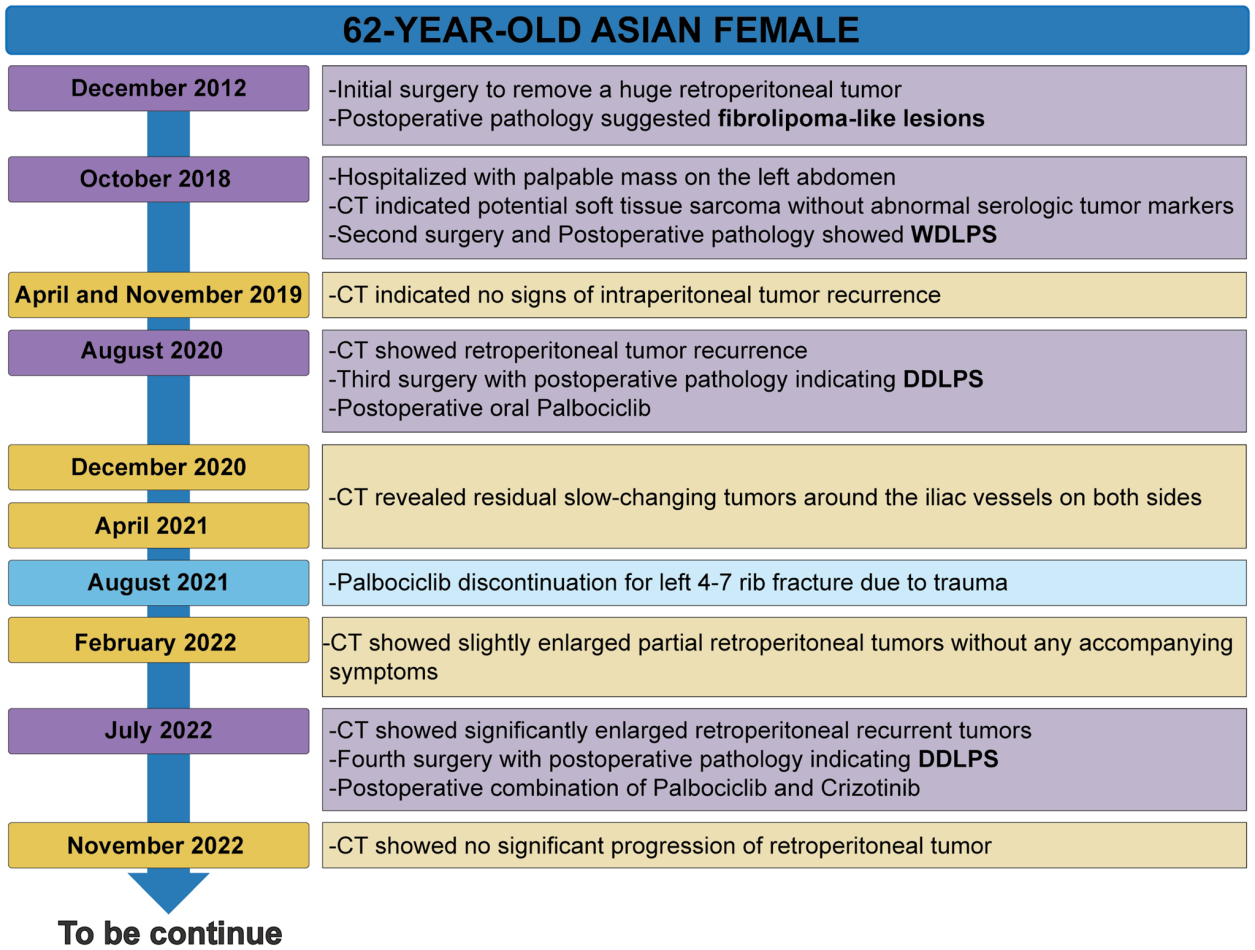
The timeline of the patient’s progress.

The patient’s timeline was presented (Figure 1), illustrating the progression of the disease, as well as the treatment interventions and follow-up activities. Her detailed medical history is as follows: A 62-year-old woman was admitted to the Seventh Affiliated Hospital of Sun Yat-sen University in October 2018 due to the discovery of an abdominal mass 2 months. In 2012, she underwent surgery at another hospital to remove a massive retroperitoneal tumor, and the postoperative pathology diagnosis was a fibrolipoma-like lesion. Abdominal computed tomography (CT) showed several masses below the left kidney and above the posterior bladder, which invaded the left kidney and ureter (Supplementary Figures S1A–D). Recurrence of the original fibrolipoma was considered, and sarcoma could not be excluded. The patient underwent a second surgical treatment, and postoperative pathology revealed: These tumors are composed of bundled or woven spindle cells and a few adipocytes of varying sizes, with “adipoblast-like” cells (Supplementary Figures S1E,F). Immunohistochemistry reveals that cancer cells express MDM2 (+) (Supplementary Figure S1G). FISH showed MDM2 gene amplification (Supplementary Figure S1H). The pathological diagnosis was well-differentiated retroperitoneal liposarcoma.

In August 2020, a follow-up CT considered liposarcoma recurrence followed by the third surgical treatment (Supplementary Figures S2A,B). The post-operative pathology revealed that mature adipose tissue and adipose cells, along with moderate-to-severe heterogeneous spindle cells, pleomorphic cells, or tumor giant cells, and scattered nuclei with enlarged, deep-stained, vacuolated cytoplasm and indentation at the nucleus margin, demonstrating pleomorphic adipocyte-like changes (Supplementary Figures S2C–F). Immunohistochemistry reveals that tumor cells are positive for MDM2 (+), CDK4 (+) and P16 (+) (Supplementary Figures S2G–I). The patient was diagnosed with DDLPS based on HE and IHC findings. The genetic sequencing results indicated the patient harbored seven somatically altered genes (Table 1). After careful consideration, we recommend Palbocinb treatment. In August of 2021, the patient discontinued Palbocinb due to the trauma.

**Table 1:** Postoperative target region sequencing results of DDLPS in 2020 and 2022.

Test items	DDLPS in 2020	DDLPS in 2022
Somatic mutations of potential clinical significance		
Copy number amplification	MDM2; CDK4; ROS1; FRS2; ESR1;	MDM2; CDK4;
Copy number deletion	ATM; CBL;	ATRX;
Gene rearrangement	–	POC1B-ROS1 (P10:R19)
Germline mutation of potential clinical significance	Negative	Negative
TMB	3.35Muts/Mb	0Mut/Mb
MSI	MSS	MSS
PD-L1	Negative (TPS <1%, CPS <1)	Negative (TPS <1%, CPS <1)

**Figure 2:**
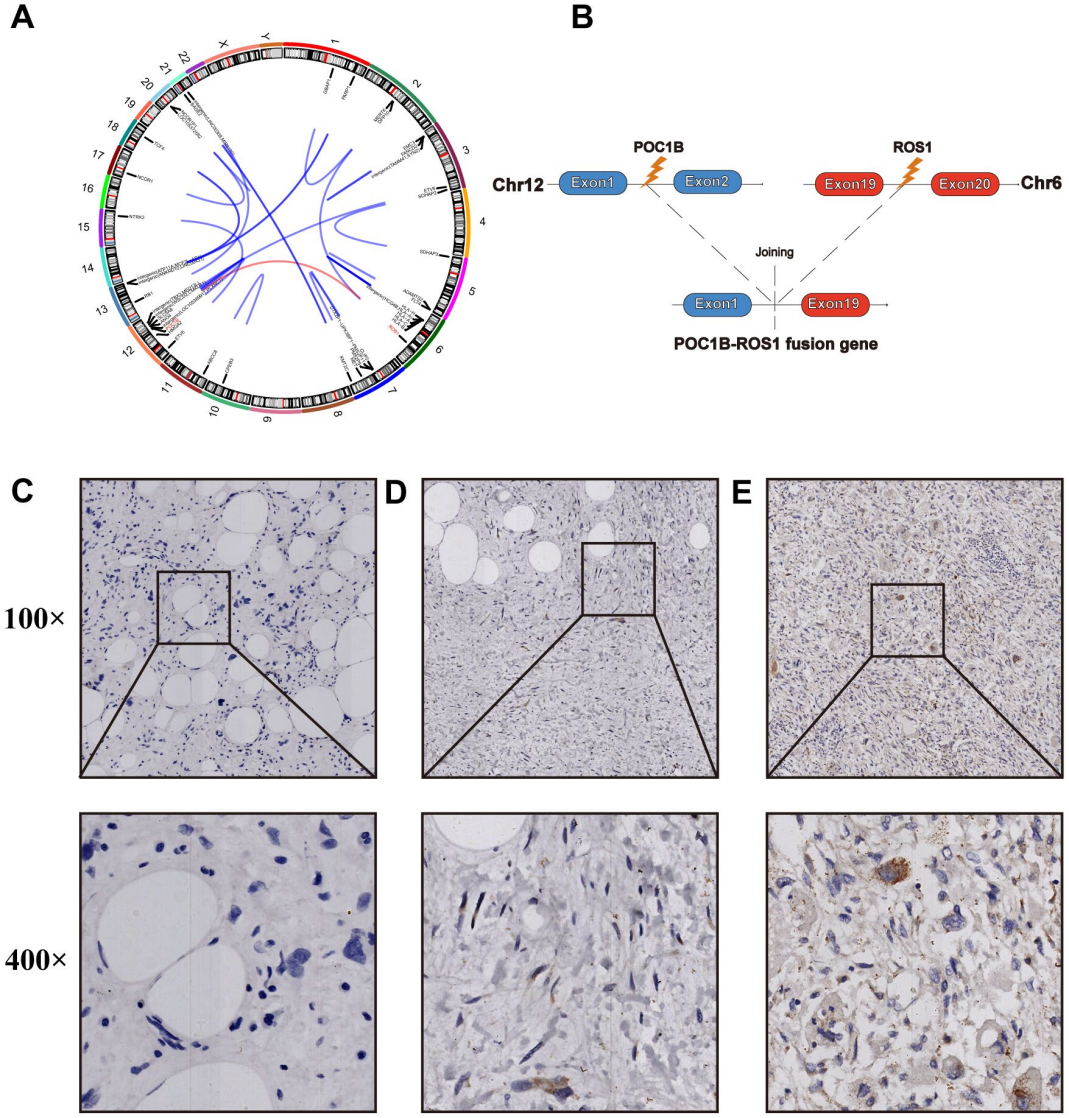
**(A)** The circos diagram shows all fusion loci from the 2022 postoperative tumor samples. **(B)** Illustration of the POC1B-ROS 1 rearrangement (the new variant is composed of POC1B exon 1 and ROS1 exons 1–19). ROS1 expression was verified by immunohistochemical (IHC) staining in different postoperative tumor specimens from 2018 **(C)**, 2020 **(D)**, and 2022 **(E)**.

In July 2022, a follow-up CT revealed the recurrent and growing tumor and a fourth abdominal surgery was performed. Histopathology and immunohistochemistry were similar to previous postoperative findings, but more heterotypic spindle cells and nuclear division were observed (Supplementary Figure S3). The genomic sequencing of the patient’s tumor tissue revealed the presence of 4 somatically mutated genes of potential clinical significance (Table 1). Sequencing data revealed more fusion sites on chromosomes 12 and 6, as well as a previously unknown POC1B::ROS1 rearrangement (P10:R19), including exons 1–19 of ROS1 and exon 1 of POC1B, with an abundance of 11.68% (Figures 2A,B). The IHC analysis revealed that early-stage tumors (WDLPS) lacked ROS1 expression, whereas DDLPS contained only a few ROS1-positive cells (Figures 2C–E). Based on the POC1B::ROS1 gene rearrangement (fusion), we elected to treat the patient with a combination of palbociclib and crizotinib medications. The patient displayed no specific adverse effects following oral administration of the drugs and this continues to be followed.

### Sample collection and DNA extraction

Unstained postoperative slices of the patient were used to obtain 1 normal tissue and 3 tumor tissues that were formalin-fixed and paraffin-embedded (FFPE) (containing WDLPS tissue from 2018, and DDLPS tissue from 2020 and 2022). Following the manufacturer’s instructions, the DNeasy Blood & Tissue Kit (Magen, China) was used to extract and purify DNA from the obtained tissues. Using a NanoDrop ND-2000 spectrophotometer, the DNA’s concentration and quality were identified. Finally, 0.8% agarose gel electrophoresis was conducted to verify the DNA’s integrity. Four DNA samples passed all quality control procedures and are available for WES analysis. Genomic DNA was fragmented by sonification followed by DNA ends repairing. Adapters were added at both ends of each fragment. Biotinylated RNA library baits and magnetic beads were mixed with the barcoded library for targeted regions selection with the Agilent SureSelect Human All Exon V6 Kit. The captured sequences were further amplified for 150 bp paired-end sequencing in the Illumina X-ten system.

### Whole-exome sequencing

#### Clean reads filtering

Quality trimming is an essential step to generate high confidence in variant calling. Raw reads would be processed to get high-quality clean reads according to three stringent filtering standards: 1. removing reads with ≥ 10% unidentified nucleotides (N); 2. removing reads with > 50% bases having phred quality scores of ≤ 20; 3. removing reads aligned to the barcode adapter.

#### Variants identification and annotation

To identify single nucleotide polymorphisms (SNPs) and insertions/deletions (INDELs), the Burrows-Wheeler Aligner (BWA) was used to align the clean reads from each sample against the reference genome with the settings “mem 4 -k 32 -M,” -k is the minimum seed length, and -M is an option used to mark shorter split alignment hits as secondary alignments (7). Variant calling was performed for multi-sample using the Genome Analysis Toolkit (GATK) (8) Unified Genotyper with local realignment, and base quality score recalibration. SNPs and INDELs were filtered using GATK’s Variant Filtration with proper standards (-Window 4, −filter “QD < 2.0 || FS > 60.0 || MQ < 40.0,” −G_filter “GQ < 20”) and those exhibiting segregation distortion or sequencing errors were discarded.

### Variant frequency

To determine the frequency of each SNP, the software tool ANNOVAR (9), was used to align and annotate SNPs or INDELs to the following database: 1000 Genomes Project,^1^ HAMAP,^2^ ESP6500,^3^ dbSNP,^4^ Kaviar.^5^

### Mutation deleteriousness

SIFT, Polyphen-2, MutationTaster, CADD, LRT, Fathmm, PROVEAN, DANN, MutationAssessor, fathmm-MKL, MetaSVM, GERP++, phyloP, phastCons, SiPhy were used to predict mutation deleteriousness and degree of locus conservation (10–24). Variants related to diseases were annotated with Clinvar,^6^ OMIM,^7^ and COSMIC70.^8^

### Structural variations identification

Structural variations (SVs) types include translocations, inversions, and insertion events, and SVs were determined by the software CREST (1.0) (25). Copy number variants (CNVs) were classified by control-freec(10.4) (26).

### Target region sequencing

The next generation high-throughput sequencing technology based on the Illumina sequencing platform was used to examine post-operative samples from the patient in 2020 and 2022. The detection can cover SNV, INDEL, CNV, and gene rearrangement in the +/−20 bp range of the target gene exon.

### Bioinformatics analysis based on the cancer genome atlas (TCGA)

Transcriptome sequencing data and corresponding clinical information were collected for sarcoma (SARC) Samples from TCGA,^9^ a total of 67 LPS patients including DDLPS (n = 64), WDLPS (n = 1), and PLPS (n = 2). Prognostic analysis was performed on the top 15 genes of CNV that gradually increased with disease progression in this patient. The survival analysis of the data for those genes was conducted using the Kaplan–Meier (KM) method (best cut-off, *p* < 0.05), and the “survival” and “survminer” packages were used for evaluation. Furthermore, the association between gene expression and survival was analyzed using Cox’s analysis with the “survival” packages. In addition, fusion gene events were identified based on multiple online fusion gene databases (FPIA (27), ChimerDB 4.0 (28), and TCGA Fusion Gene Database (29)), and further prognostic analysis was performed with the KM method.

## Results

### Identification of somatic mutation from WDPLS and DDPLS

Supplementary Tables S1–S5 provides a detailed listing of SNP and INDELs data for different samples. The mutation patterns of WDLPS and DDLPS were similar, and we noticed that C: G > T: A was the most prevalent somatic translocation (Supplementary Table S6).

**Figure 3:**
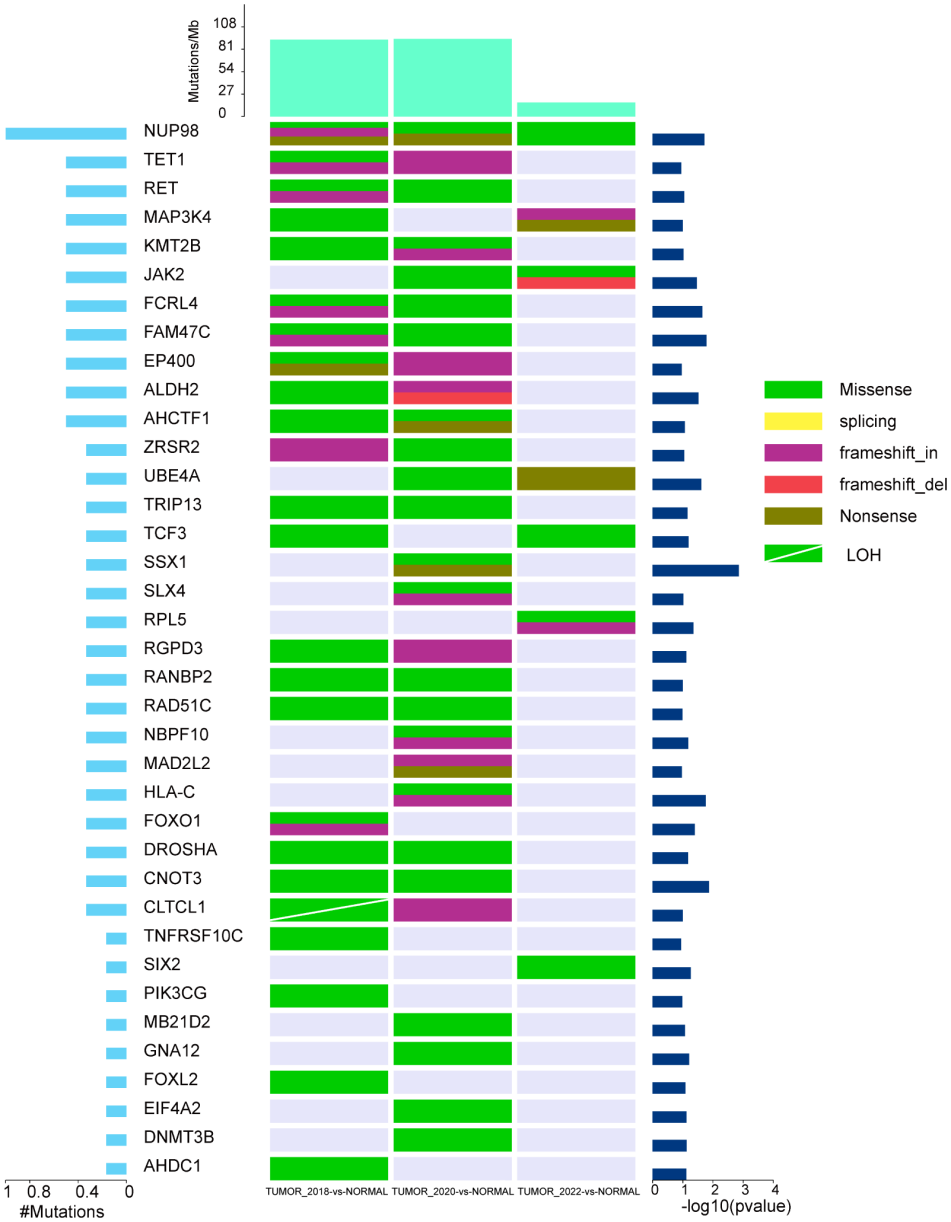
The landscape of high-frequency mutant cancer driver genes in WDLPS and DDLPS at different times. The middle panel shows the somatic mutations by sample (column) and gene (row). The histogram at the top shows the number of mutations accumulated in each individual sample, and mutation types are marked with different colors.

We observed 11,198 SNPs in the WDLPS by comparing normal tissues, of which 2,306 were nonsynonymous somatic mutations. The DDLPS identified 7,308 SNPs, 1,542 of which were non-synonymous somatic mutations (Supplementary Table S7). Moreover, we discovered 3,920 somatic INDELs in the WDLPS and 5,679 in the DDLPS. Considering somatic SNPs and INDELs, by comparing normal samples and using MuSic to identify genes with high mutation frequency in tumor samples, 422 high-frequency mutated genes were confirmed in WDLPS, and 471 high-frequency mutated genes were confirmed in DDLPS, approximately 50%, for a total of 242 high-frequency mutated genes shared by WDLPS and DDLPS (Supplementary Tables S8, S9). We also found 229 genes exclusively mutated in DDLPS. In addition, the high-frequency mutated genes in DDLPS were reduced after treatment with the Palbociclib drug (Supplementary Table S10). We discovered 37 mutated cancer driver genes in WDLPS and DDLPS by comparing data from several databases (Cancer Gene Census, CGC, http://cancer.sanger.ac.uk/census; MDG125; SMG127; CDG291; NCG, http://ncg.kcl.ac.uk/canonical_drivers.php; OncoKB; https://www.oncokb.org/cancerGenes). During the progression of WDLPS to DDLPS, the mutation types of 37 cancer driver genes increased, but they reduced dramatically following Palbociclib treatment (Figure 3).

### Differences in genomic CNVs between WDLPS and DDLPS

**Figure 4:**
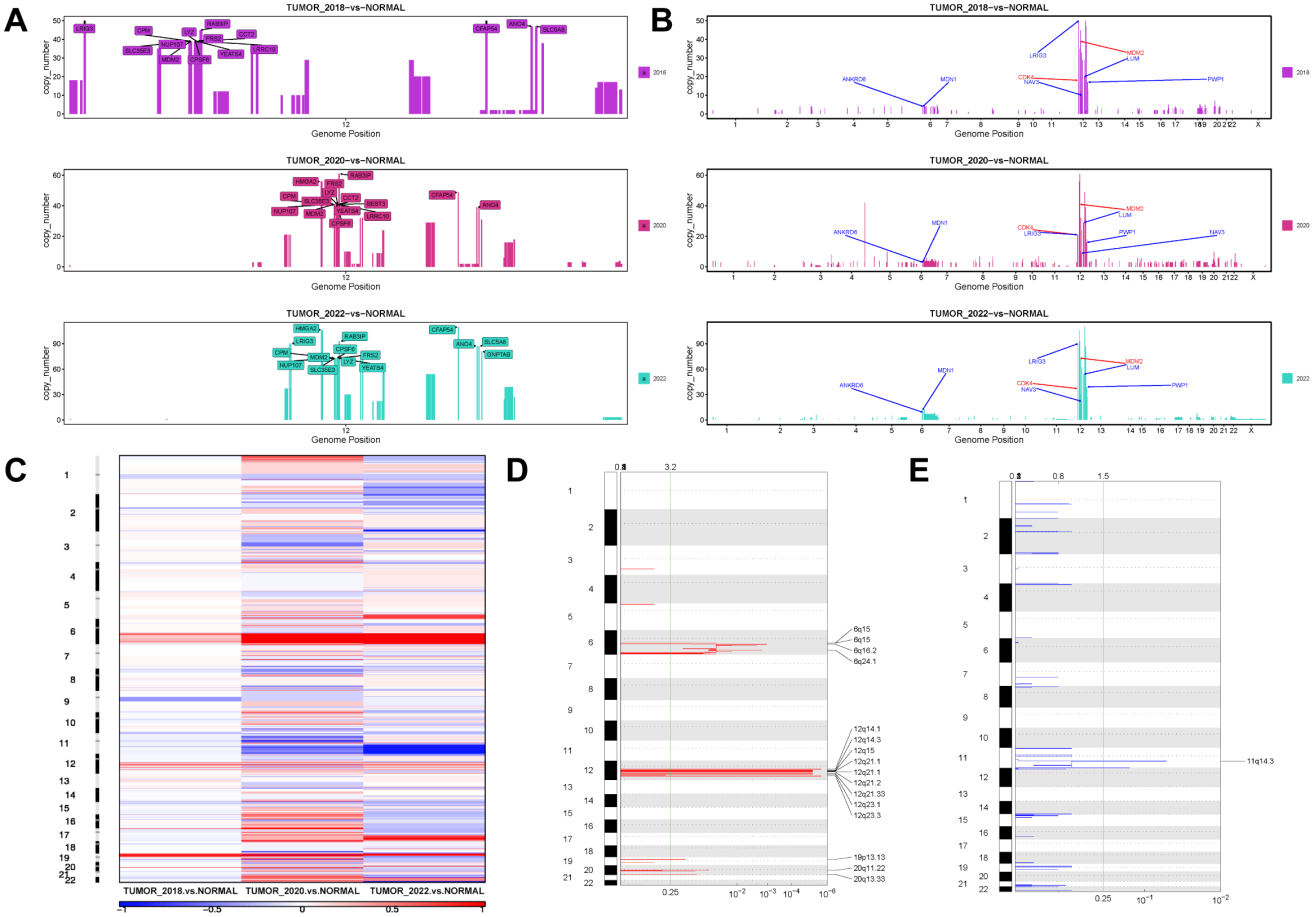
The landscape of CNV in WDLPS and DDLPS at different times. **(A)** The top 15 genes with copy number amplification on the long arm of chromosome 12 (12q) at different times. **(B)** Amplification profiles on chromosomes of six cancer driver genes (blue) and diagnostic genes (red). **(C)** A heat map of the distribution of high-frequency CNV in different samples GISTIC 2.0 plot of recurrent focal gains **(D)** and losses **(E)** Chromosomes are represented along the vertical axis; *q* values are marked along the horizontal axis. The green lines mark the cutoff for the significance threshold (*q* = 0.25).

CNVs were identified in 155 and 250 genomic regions, respectively, in WDLPS and DDLPS, including copy number increase, decrease, and loss of heterozygosity (LOH) (Supplementary Table S11). Analysis of copy numbers showed that WDLPS had several significant copy number increases in the long arm of chromosome 12 (12q), while copy numbers of other chromosomes were only slightly elevated (Supplementary Table S12). For these amplified 12q regions, the most notable amplifications included LRIG3 at 12q14.1, CFAP54, ANO4, and SLC5A8 at 12q23.1, MDM2, YEATS4, and CPM at 12q15, etc. (Figure 4A). DDLPS had high levels of chromosomal instability/copy number alterations and also shows increased copy number on chromosome 12q with a greater concentration of high-level amplification regions (Supplementary Table S13). As the disease progresses, these regions have higher levels of copy number increase, such as CPM (73 copies), HMGA2 (106 copies), and MDM2 (73 copies) amplification, suggesting clonal evolution and selection for higher levels of amplification of these genes (Supplementary Tables S14, S15). Comparing these amplified genes to the cancer driver genes in the database, we identified six amplified cancer driver genes, with LRIG3 showing the highest level of amplification (Figure 4B). We observed that most genes with high copy number amplification indicated poor prognosis in patients using the expression matrix and clinical data of lipomatous neoplasms patients in the TCGA-SARC cohort (30) (Supplementary Figure S4), and Univariate Cox hazard analysis revealed that SLC35E3 could be an independent prognostic factor in patients with LPS (Supplementary Table S16). CNV results were converted to segment format by DNAcopy and then analyzed by GISTIC2 for high-frequency Somatic CNV. Other regions of CNV gain included 6q15, 6q16.2, 6q24.1, 12q14.3, 12q21.1, 12q21.2, 12q21.33, 12q23.3, 19p13.13, 20q11.22,20q13.33, with the deletion mainly concentrated in 11q14.3 (Figures 4C–E).

### TMB, MSI, and HRD evaluation in WDLPS and DDLPS

Tumor Mutational Burden (TMB), defined as the number of somatic mutations in the CDS region per megabase (MB) of the longest transcript sequence, differs amongst tumors (31). TMB can be used to predict immunotherapy efficacy and potentially increase the number of patients for immune checkpoint inhibitors (ICIs) treatment. TMB was calculated using somatic SNVs and somatic INDELs, and the results indicate that TMB decreases as cancer progresses (Supplementary Table S17). In addition, short tandem repeat (STR) or simple sequence repeat (SSR) length changes caused to deficiencies in the mismatch repair (MMR) system are known as Microsatellite Instability (MSI), which can be considered an important biomarker for solid tumor adjuvant treatment (32). Both the WDLPS and DDLPS msing scores are less than 0.2 (Supplementary Table S18), suggesting microsatellite stability (MSS). Homologous recombination (HR) is a highly conserved process that plays an important role in DNA repair, DNA replication, meiotic chromosome segregation, and telomere maintenance. However, when DNA damage occurs, homologous recombination deficiency (HRD) occurs if the DNA damage cannot be repaired properly by the homologous recombination repair (HRR) pathway. HRD can be employed as a biomarker to guide the clinical application of platinum-based chemotherapy treatments and PARP inhibitors (33). Considering the results of the loss of heterozygosity (LOH), telomeric allelic imbalance (TAI), and large-scale state transition (LST), the HRD ratings were all less than 42 (Supplementary Table S19), indicating that both WDLPS and DDLPS were HRD-negative.

### Fusion gene analysis based on bioinformatics

Although ROS1-related fusion gene events have been reported in a variety of cancers and their impact on tumor progression and prognosis, ROS1-related fusion gene events are relatively rare in WDLPS/DDLPS. TCGA transcriptome data analysis and online fusion gene databases showed that ROS1-related fusion gene mutations were only identified in glioblastoma (GBM) and lung adenocarcinoma (LUAD) (Supplementary Tables S20–S22). From three databases, fusion events for WDLPS/DDLLPS patients were collected. Intersecting the top 10 genes with the greatest fusion mutation frequency in different databases revealed that FRS2, PTPRR, CPM, DNM3, TMTC2, TRHDE, and RAB3IP had the highest mutation frequencies (Supplementary Tables S23–S28). Most of these genes were located on chromosome 12, indicating genomic instability on chromosome 12 in LPS patients, and Kaplan–Meier survival analysis showed that only RAB3IP-related fusion gene events were associated with poor prognosis (Supplementary Figure S5). Future studies need larger cohorts to characterize the association between fusion genes and clinical characteristics in WDLPS/DDLPS.

## Discussion

ALT/WDLPS, the most common pathological subtype of STS, accounts for approximately 40–45% of all cases (34). ALT refers to tumors that are deep-seated extremities and can be completely removed by surgery, while WDLPS refers to tumors that develop in deep, central anatomic areas and necessitate total excision, sometimes in conjunction with resection of adjacent tissues (35). ALT/WDLPS and DDLPS tend to arise in the extremities and retroperitoneum, and more rarely in the head and neck region, mediastinum, and paratesticular region (36). Up to 90% of DDLPS arise *de novo*, and the remainders occur as recurrences of previous WDLPS (37). Fibrolipoma is a benign neoplasm of common mesenchymal origin, and to date, it has rarely been reported to develop malignant lesions (38). In this case, the patient had a retroperitoneal mass resection in another hospital in 2012, and the preliminary postoperative pathology revealed a fibrolipoma-like lesion. Both the patient’s initial and recurring symptoms were slowly enlarging abdominal masses. She was pathologically diagnosed with DDLPS in our hospital after surgery in August 2020, 8 years after the first operation. Given the average interval of 7.7 years for recurrence of WDLPS progression to DDLPS (39), in addition to the sometimes insignificant histocytic heterogeneity of WDLPS and the difficulty in detecting adipoblasts and spindle cells in the fibrous septum (40), it is reasonable to speculate that there was a pathological misdiagnosis after surgery in 2012, but the lack of histological sections prevented us from further confirmation. When diagnosing deep soft tissue tumors, clinicians and pathologists should consider the possibility of WDLPS when the histological manifestations are mild, contain adipose tissue, and have a mass > 5 cm in diameter, especially the recurrent mass containing adipose tissue.

It is frequently tricky to appropriately diagnose WDLPS and DDLPS preoperatively based on clinical symptoms and imaging; therefore, postoperative pathological diagnosis is of utmost importance. WDLPS is separated pathologically into adipocytic (lipoma-like), sclerosing, and inflammatory subtypes, with the lipoma-like subtype being the most common (34). Microscopically, lipoma-like WDLPS is composed of mature adipocytes and varying numbers of adipoblasts that are multi- or single-vesicular with irregular nuclei, homogeneous chromatin, and transparent cytoplasm (36). In the septum, many spindle cells with hyperchromatic nuclei and an irregular form were identified (41). On occasion, heterogenic components like cartilage, bone, smooth muscle, and striated muscle are discovered. DDLPS can be observed microscopically as dedifferentiated regions in addition to WDLPS characteristics. The dedifferentiated components include non-adipogenic, adipogenic, and heterogenic differentiation. Typically, the boundary between differentiated and dedifferentiated regions is relatively sharp. Infrequently, the transition is gradual, with a mixture of both. From the perspective of molecular expression profiles, the majority of WDLPS/DDLPS immunohistochemically express MDM2 and CDK4 (97 and 92%, respectively), and marker expression correlates strongly with gene amplification status (42). The combination of CDK4, MDM2, and p16 can help distinguish WDLPS and DDLPS from other adipocytic tumors in the differential diagnosis (43), and FISH assessment of MDM2 amplification status can help differentiate WDLPS from lipomas and DDLPS from pleomorphic sarcomas and spindle cell sarcomas (44, 45). This patient had a typical lipoma-like WDLPS with a dedifferentiated component (fibrosarcoma-like tissue) that proceeded to DDLPS after several recurrences, according to the patient’s medical history, microscopic appearance, immunohistochemistry, and FISH test results.

As sequencing technology has advanced, the molecular anomalies of WDLPS and DDLPS have been increasingly uncovered. Both WDLPS and DDLPS have high levels of chromosome 12q13-15 amplification (46), including CDK4, CPM, HMGA2, CPM, SAS/TSPAN31, YEATS4 (47, 48), and overexpression of MDM2 as the key driver gene of 12q amplification, which is the initiating factor of WDLPS/DDLS carcinogenesis (49). The profiles of gene amplification in the 12q13-15 area differed significantly between WDLPS and DDLPS, with DDLPS exhibiting more significant levels of amplification than WDLPS. In addition, DDLPS demonstrated amplification of other chromosomal regions, particularly at 1p32 and 6q23, with 24% of DDLPS exhibiting 1q32.2 (JUN) amplification (50). Similarly, whole-exome sequencing of WDLPS and DDLPS from this patient showed previously reported amplified regions and corresponding genes. Amplification of other chromosomal regions (6q15, 6q16.2, 6q24.1, 19q13.13, 20q11.22, 20q13.33) and copy number decrease in 11q14.3 were also identified. By comparing DDLPS to WDLPS, it was found that DDLPS had significantly more mutated genes and higher levels of chromosomal amplification; moreover, after treatment with Palbociclib, DDLPS had significantly fewer mutated genes but continued to have higher levels of chromosomal amplification, which may have contributed to this patient’s relapse. The efficacy of immunotherapy in liposarcoma is currently poorly understood. Early clinical trials including Pembrolizumab and Nivolumab with/without CTLA-4 checkpoint inhibitors found that only a tiny percentage of LPS patients responded to treatment (51, 52). Similarly, sensitivity analyzes for immunotherapy, including PD-L1 expression levels, TMB, and MSI analyzes, have been performed, suggesting that DDLPS may not be amenable to immunotherapy and that more immunotherapy sensitivity testing for different LPS subtypes is necessary.

To further delay disease progression, postoperative combination drug therapy needs to be considered. However, because patients with DDLPS/WDLPS have a response rate of 11–24% to chemotherapy with chemotherapy-related toxicities, it is not routinely recommended unless the patient is symptomatic or has disease-related complications (53, 54). Similarly, neoadjuvant/adjuvant radiotherapy did not show a significant survival benefit (55). Better alternatives include numerous new oral targeted medicines with comparatively lower hematological toxicity. Current research is focused on the development of new drugs targeting WDLPS/DDLPS key driver mutated genes such as CDK4 and MDM2. Two generations of compounds have been created that reactivate TP53 by inhibiting the MDM2-TP53 connection, as MDM2 is a nuclear phosphoprotein that inhibits the TP53 pathway (56). Although preclinical research has shown strong tumor suppressor effects, early clinical trials revealed that MDM2-TP53 inhibitors elicited only partial responses in a minority of patients, with the majority of patients experiencing at least one adverse event (52). To understand the clinical benefits of MDM2-TP53 inhibitors, additional research is required, and it may be necessary to develop new MDM2 inhibitors or combinations with other medications. CDK4 is a cyclin-dependent kinase that is activated by binding to D-type allosteric cyclins (CCND) (57). It participates in the retinoblastoma (RB) pathway, which regulates the cell cycle and promotes cancer. Multiple laboratory and clinical studies have confirmed that CDK4 suppresses the evolution of liposarcoma by negatively regulating the RB pathway (58). Palbociclib is an oral CDK inhibitor that has been approved by the Food and Drug Administration (FDA) for the combination treatment of patients with ER+/HER2- advanced breast cancer. Numerous studies have revealed that patients with CD4-amplified WDLPS and DDLPS have a poor prognosis (59–61), with over 90% of WDLPS and DDLPS showing CDK4 amplification. A clinical phase 2 study of palbociclib (PD0332991) showed that 60 patients with WDLPS/DDLPS treated with palbociclib had a 12-week progression-free survival rate of 57.2% and a median PFS of 17.9 weeks (62), suggesting that palbociclib could be a treatment option for patients with CDK4-amplified WDLPS/DDLPS. The clinical trials with Palbociclib for second-line therapy of overexpressed CDK4 sarcoma are under underway [NCT03242382]. After progression to DDLPS, we performed two targeted sequencings on her, both of which suggested many gene amplifications, including CDK4 and MDM2. In the postoperative DDLPS specimen from the patient in 2022, targeted sequencing results revealed the occurrence of ROS1-related fusion genes, and IHC analysis confirmed the expression of ROS1 protein in a small subset of tumor cells. ROS1 is located on chromosome 6q22.1 and is associated with several downstream signaling pathways involved in cellular differentiation, proliferation, growth, and survival. It is noteworthy that known ROS1-related fusion genes in retroperitoneal tumors include FRK::ROS1 and VGLL2::ROS1 (63), both located on chromosome 6, whereas POC1B is located on chromosome 12q21.33. The identification of this fusion gene pair further emphasizes the potential instability and interaction between chromosomes 12 and 6 in DDLPS. However, due to the limited functional analysis available for these ROS1-related fusion genes, the oncogenic potential of these specific chimeric proteins and which patients may benefit from anti-ROS1 tyrosine kinase inhibitor therapy cannot be determined. Further evaluation of the combination of Palbociclib and Crizotinib is still required.

## Conclusion

Although ALT/WDLPS and DDLPS represent a spectrum of a single disease entity with similar genetic abnormalities, Careful microscopic inspection of histocyte morphology combined with immunohistochemistry and FISH may avoid misdiagnosis of WDLPS, which is sometimes difficult to discriminate from benign and malignant adipocytic tumors and fibrous tumors. Due to the high recurrence rate of retroperitoneal WDLPS and DDLPS, it is important for accurate diagnosis and regular follow-up of these diseases. It is essential to select the most effective targeted medication based on the post-operative targeted sequencing results along with the patient’s condition to halt the progression of cancer. However, additional research is required in the future to reveal the molecular abnormalities of WDLPS and DDLPS and to develop drugs that are corresponding to those abnormalities.

## Data availability statement

The original contributions presented in the study are included in the article/Supplementary materials, further inquiries can be directed to the corresponding authors.

## Ethics statement

The studies involving human participants were reviewed and approved by The Medical Ethics Committee of the Seventh Affiliated Hospital of Sun Yat-sen University. The patients/participants provided their written informed consent to participate in this study. Written informed consent was obtained from the individual(s) for the publication of any potentially identifiable images or data included in this article. Written informed consent was obtained from the participant/patient(s) for the publication of this case report.

## Author contributions

CZ and MH designed, organized, and supervised the study. ZZ, YS, TM, and JX: data collection. ZZ and XC: manuscript writing. CZ: manuscript editing. All authors contributed to the article and approved the submitted version.

## Funding

This study was supported by the Guangdong Provincial Key Laboratory of Digestive Cancer Research (No. 2021B1212040006), Sanming Project of Medicine in Shenzhen (No. SZSM201911010), and Shenzhen Key Medical Discipline Construction Fund (No. SZXK016).

## Conflict of interest

The authors declare that the research was conducted in the absence of any commercial or financial relationships that could be construed as a potential conflict of interest.

## Publisher’s note

All claims expressed in this article are solely those of the authors and do not necessarily represent those of their affiliated organizations, or those of the publisher, the editors and the reviewers. Any product that may be evaluated in this article, or claim that may be made by its manufacturer, is not guaranteed or endorsed by the publisher.

## Footnotes

^1^http://www.internationalgenome.org/

^2^http://hamap.expasy.org/

^3^https://esp.gs.washington.edu/drupal/

^4^https://www.ncbi.nlm.nih.gov/projects/SNP/

^5^http://db.systemsbiology.net/kaviar/

^6^http://www.clinvar.com/

^7^https://www.omim.org/

^8^http://cancer.sanger.ac.uk/cosmic

^9^https://portal.gdc.cancer.gov/
