# Supplementary figures and images for: Whole exome sequencing of well-differentiated liposarcoma and dedifferentiated liposarcoma in older woman: a case report
Source: Front Med (Lausanne). 2023 Aug 15;10:1237246. doi: 10.3389/fmed.2023.1237246 (PMC10464618; doi:10.3389/fmed.2023.1237246)

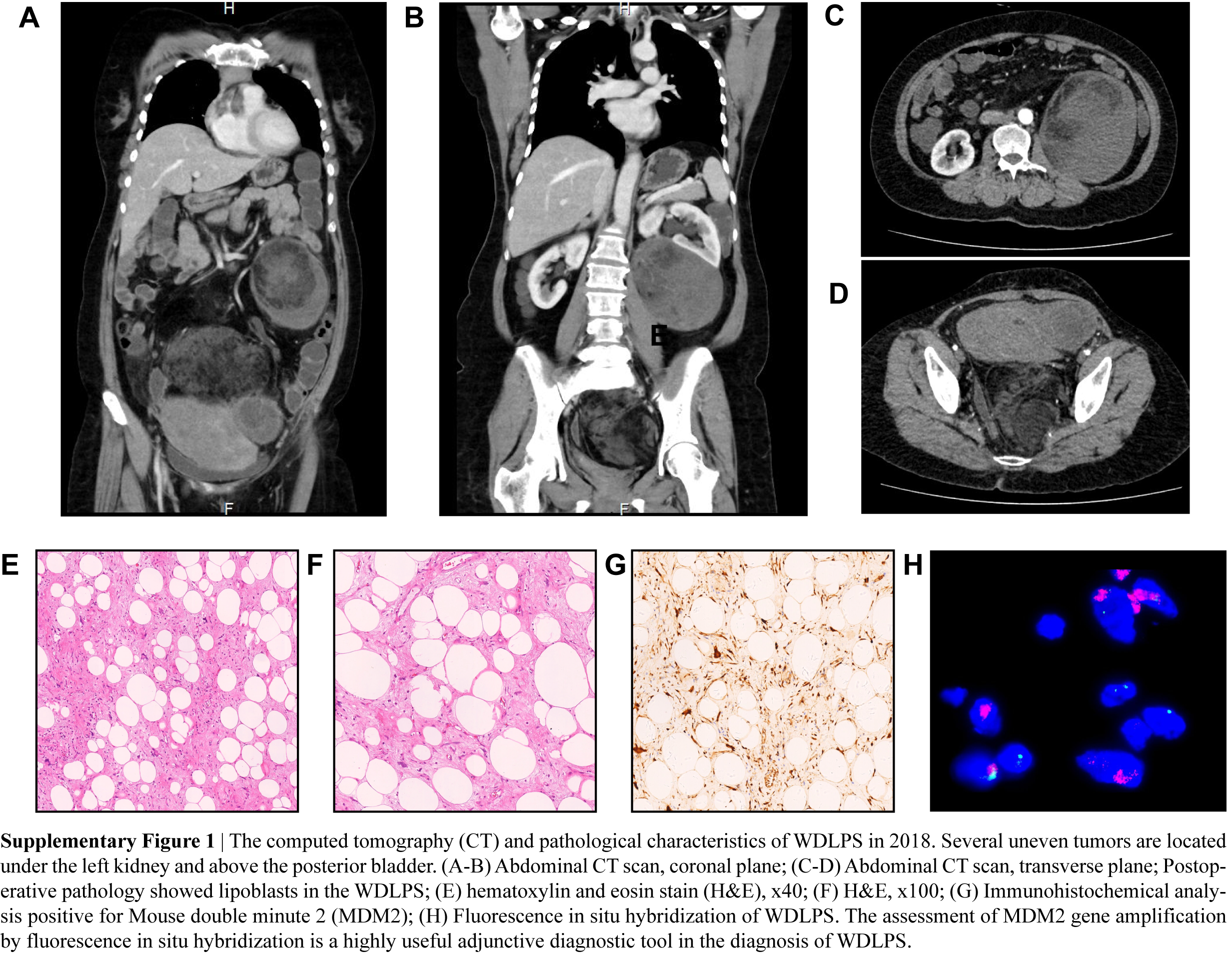

Supplement: Supplementary file 1 [file Image_1.TIF]

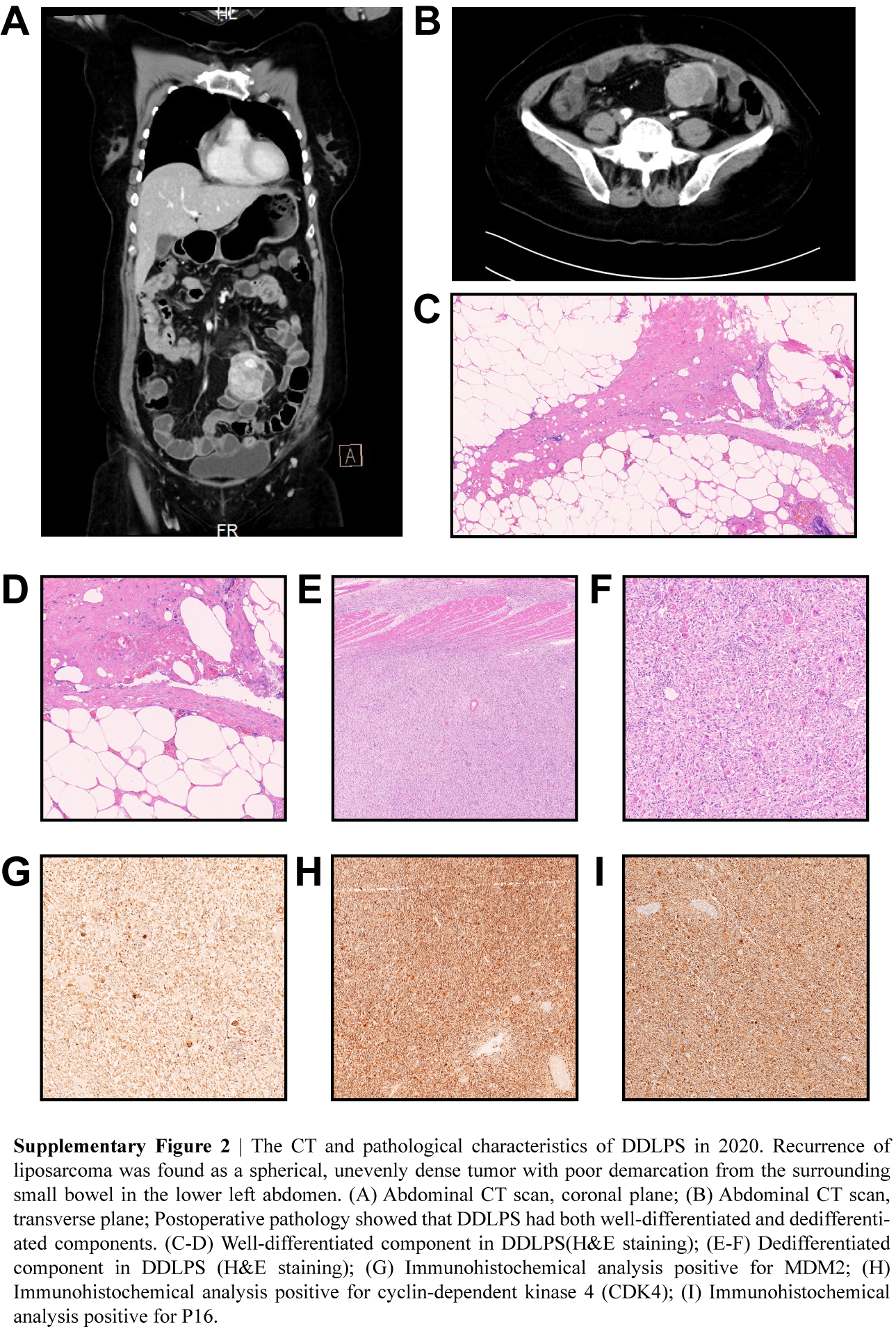

Supplement: Supplementary file 2 [file Image_2.TIF]

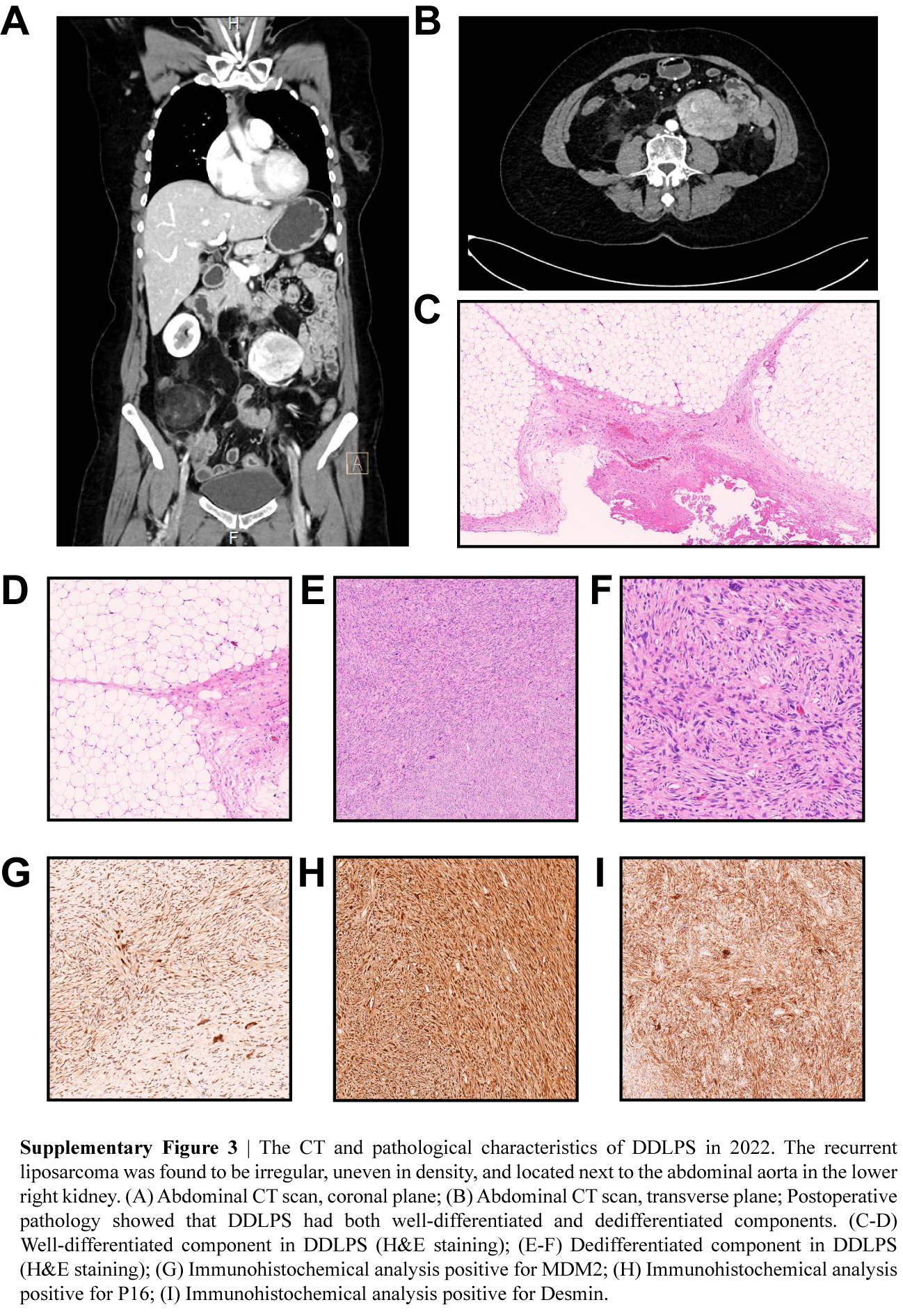

Supplement: Supplementary file 3 [file Image_3.TIF]

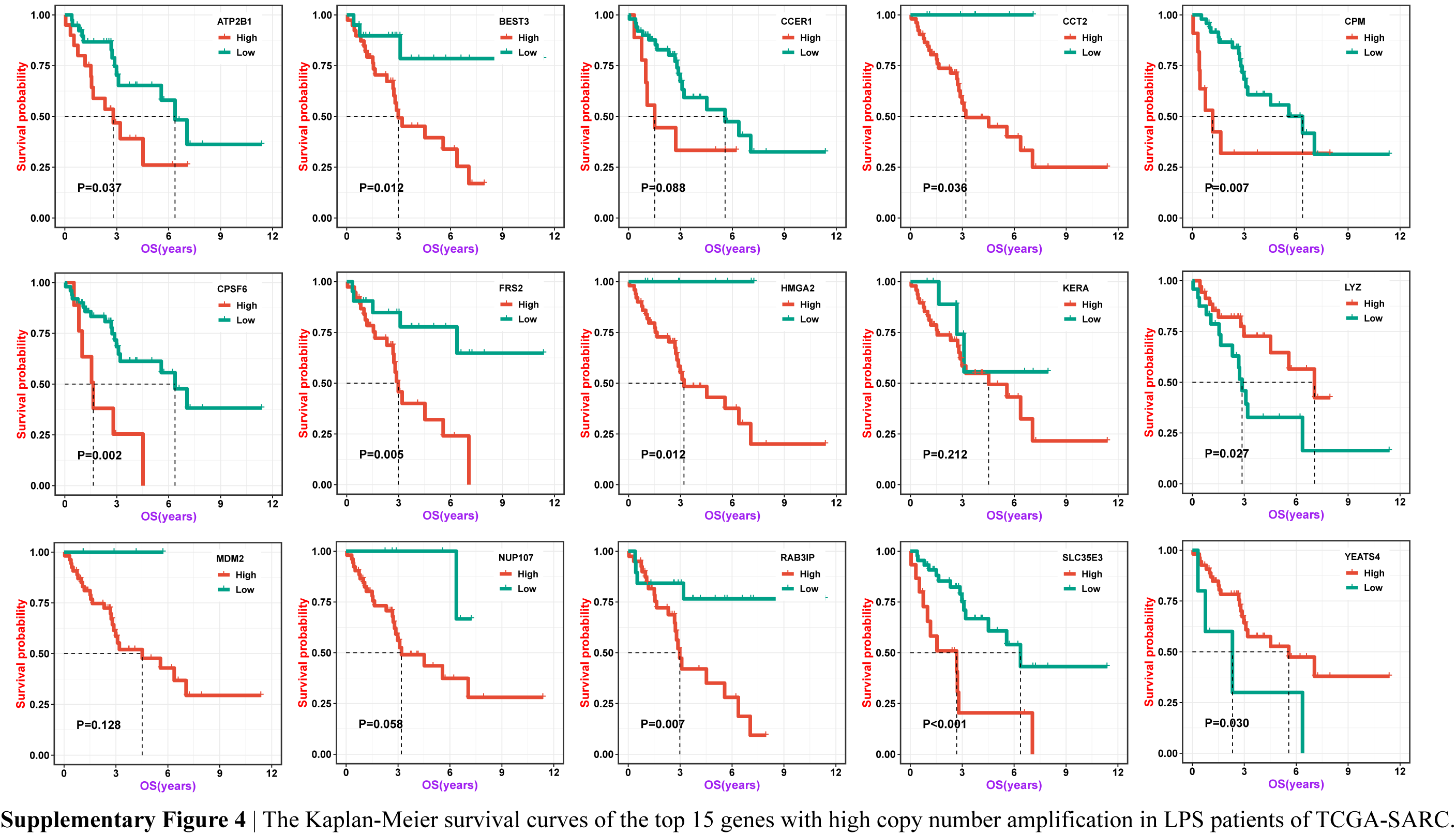

Supplement: Supplementary file 4 [file Image_4.TIF]

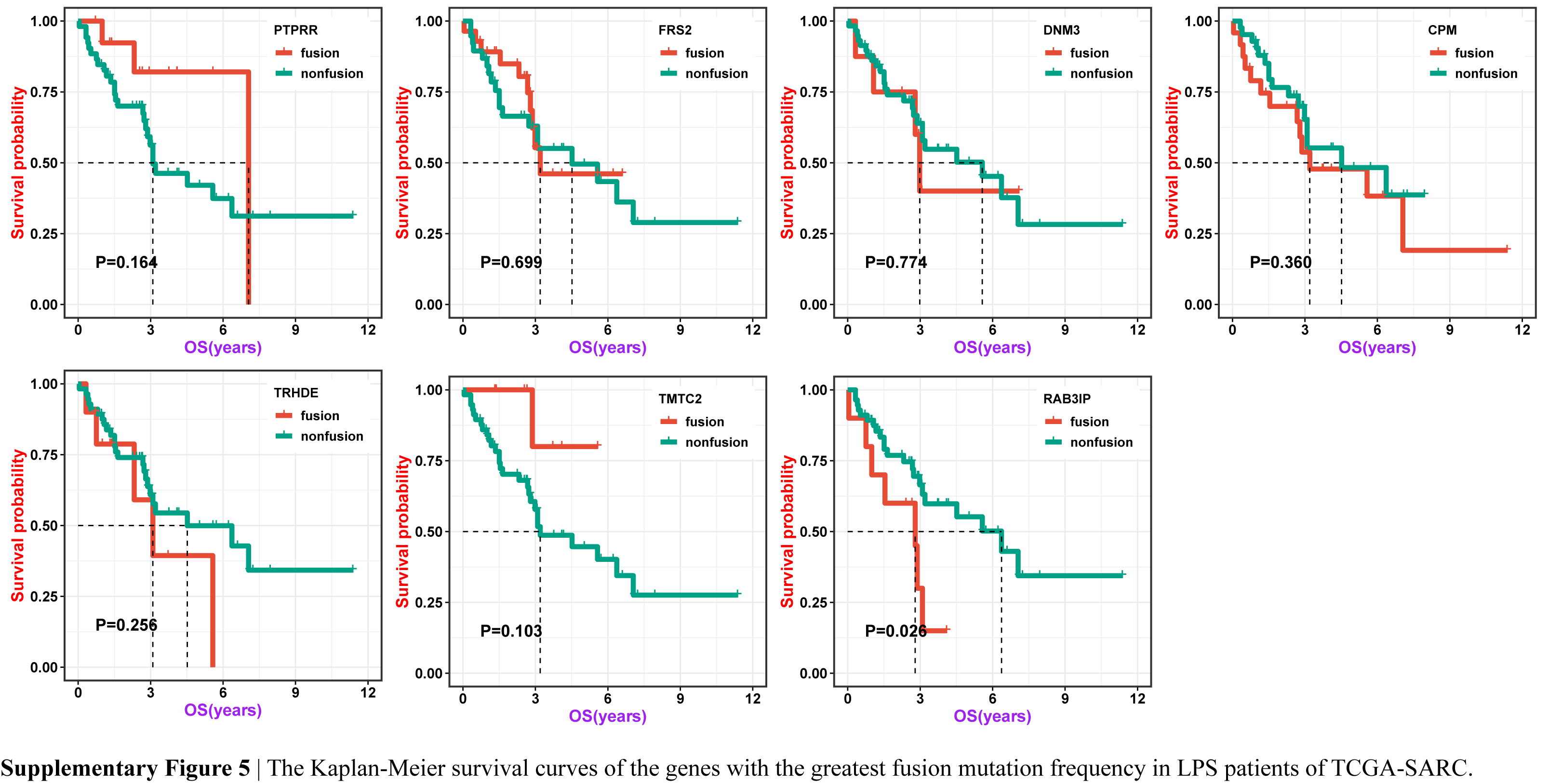

Supplement: Supplementary file 5 [file Image_5.TIF]
